# Phenological Development, Environmental Effects, and Selection of Soybean Lines from Crosses with Contrasting Relative Maturity Groups

**DOI:** 10.3390/biology15171464

**Published:** 2026-08-28

**Authors:** Eryadison Flávio Bonifacio de Araujo, Dardânia Soares Cristeli, Alyce Carla Rodrigues Moitinho, Filipe Manoel Ferreira, Davi Souza Della Libera, Laura Pinto Rodrigues, Matheus Siqueira de Oliveira, Jardel da Silva Souza, Luis Fernando Alliprandini, Glauco Vieira Miranda, Sandra Helena Unêda-Trevisoli

**Affiliations:** 1 School of Agricultural and Veterinary Sciences (FCAV), São Paulo State University “Júlio de Mesquita Filho” (UNESP), Jaboticabal 14884-900, SP, Brazil; dardania.cristeli@unesp.br (D.S.C.); acr.moitinho@unesp.br (A.C.R.M.); davi.souza@unesp.br (D.S.D.L.); laura.p.rodrigues@unesp.br (L.P.R.); matheus.siqueira@unesp.br (M.S.d.O.); jardel.souza@unesp.br (J.d.S.S.); shu.trevisoli@unesp.br (S.H.U.-T.); 2 Department of Forest Sciences, ”Luiz de Queiroz” College of Agriculture (ESALQ), University of São Paulo (USP), Piracicaba 13418-900, SP, Brazil; filipeferreira@usp.br; 3 Bayer Crop Science, Rolândia 86600-000, PR, Brazil; luis.alliprandini@bayer.com; 4 Department of Agronomy, Federal University of Technology Paraná (UTFPR), Santa Helena 85892-114, PR, Brazil; glaucovmiranda@utfpr.edu.br

**Keywords:** environmental covariates, *Glycine max*, mixed models, phenological cycle, REML/BLUP, genetic variability

## Abstract

Soybean is the main oilseed crop cultivated worldwide and is important for human and animal nutrition. The duration of flowering, the reproductive period, and maturity is determined by genetic factors and influenced by environmental conditions, including temperature, photoperiod, precipitation, soil type, and crop management, which affect yield and regional adaptation. However, important gaps remain in understanding how soybean lines from different relative maturity groups respond to these conditions, particularly regarding the effects of temperature and precipitation on phenological development across growing seasons. Therefore, this study aimed to characterize the behavior of soybean populations and the environmental effect on the phenological cycle of the crop. Two populations were developed from biparental crosses with contrasting RMG values among the parental cultivars. The E2 population, comprising 150 lines, originated from the cross BMX Potência RR (RMG 6.7) × BMX Energia RR (RMG 5.3), whereas the E3 population, comprising 80 lines, originated from BRS 245 RR (RMG 7.3) × BRS 278 RR (RMG 9.4). The populations were evaluated over four growing seasons using an augmented block design, and the traits were analyzed using mixed models with environmental covariates. REML/BLUP methodology was used to estimate genetic parameters and predict genotypic values, enabling the selection of dual-purpose lines combining earliness and high yield. Higher temperatures and lower precipitation were associated with shorter phenological periods, whereas higher precipitation and lower temperatures were associated with longer periods. Population E2 showed more consistent associations with temperature and precipitation, whereas Population E3 showed weaker or nonsignificant associations for some traits, particularly NDM. These population-specific responses may be relevant for the selection of soybean lines in breeding programs.

## Simple Summary

Soybean cultivars differ in how quickly they grow, flower, form seeds, and reach maturity. These differences are important because they influence when the crop can be harvested and how well it performs under different growing conditions. In regions where temperature and rainfall vary between seasons, understanding these differences can help breeders choose soybean lines that are better suited to each environment. In this study, two soybean populations with contrasting maturity patterns were evaluated across four growing seasons. Warmer and drier conditions were generally associated with shorter crop cycles, whereas cooler and wetter conditions were associated with longer cycles. Population E2 showed more consistent responses to environmental variation, while Population E3 showed weaker or nonsignificant responses for some traits, particularly the total cycle. These findings provide useful information for soybean breeding programs by showing how different populations may respond to environmental variation.

## 1. Introduction

Soybean (*Glycine max* (L.) Merrill) is the main oilseed crop cultivated worldwide, with a major impact on protein and vegetable oil supply, being important for human and animal nutrition as well as bioenergy production [1]. In the 2025/2026 growing season in Brazil, soybean production is estimated at 180.13 million tons [2], maintaining the country as the world’s largest producer. Because of its economic importance and wide cultivation across diverse environments, agronomic traits related to the phenological cycle, such as number of days to flowering and maturity, have gained prominence in plant breeding programs. Genetic advances in soybean have been accompanied by changes in cycle duration, resulting in the development of more productive cultivars adapted to environmental conditions [3]. For this purpose, it is essential to classify cultivars according to their relative maturity group (RMG), allowing their proper allocation to different growing environments, since phenological maturity is strongly influenced by environmental factors such as latitude, altitude, and climate [4] in each region.

RMG is a widely used classification of the phenological cycle of soybean cultivars based primarily on their responses to photoperiod and temperature, which regulate flowering and maturity [4]. The RMG system is an adaptation of the North American system, grouping genotypes into 13 classes (000, 00, 0, I, II, III, IV, V, VI, VII, VIII, IX, and X) representing adaptation ranges, from extremely early materials (group 000, high latitudes) to the latest ones (group X, latitude 0°) [5]. In Brazil, this classification was converted into a continuous numerical scale ranging from 5.0 (early cultivars) to 10.0 (late cultivars), facilitating regional cultivar placement and breeding cross planning [4]. RMG also helps define suitable sowing windows and growing environments for each cultivar. Each adaptation zone is expected to meet the developmental requirements of particular maturity groups. When cultivars are grown outside their optimal sowing window or adaptation zone, however, their phenological cycle may be shortened or extended beyond the expected pattern [6].

From a biological perspective, this classification is associated with genetic differences underlying soybean phenological development. According to previous studies [7,8,9], genetic advances have enabled the identification of important loci and regulatory pathways associated with soybean flowering and maturity, including E-series genes, the J gene, circadian clock components, FT-like genes, and several QTLs. For example, dominant alleles at the E1, E2, E3, E4, E7, E8, and E10 loci delay flowering, whereas dominant alleles at E6, E9, E11, and J promote earlier flowering [7]. These loci influence the transition from vegetative to reproductive development, the duration of the reproductive period, and the time required to reach maturity. Thus, differences among genotypes in RMG represent not only agronomic variation, but also differences in the genetic control of phenology under different environmental conditions.

In addition to genetic factors inherent to each RMG, phenotypic expression is strongly influenced by environmental variables. In soybean, the main environmental effects related to changes in phenological behavior are temperature, photoperiod, and precipitation [10], as well as soil type and agricultural practices [11], also affecting yield and seed composition-related traits [12]. Considering these complex interactions, appropriate statistical methodologies are essential. Mixed models via REML/BLUP are particularly useful in plant breeding because they can handle unbalanced data, estimate variance components, predict genetic values with high accuracy, and incorporate environmental covariates into the analysis [13].

Although these approaches have been investigated separately, few studies have integrated advanced biparental populations, repeated evaluations across growing seasons, stage-specific environmental exposure, and REML/BLUP-based selection. This integration addresses an important gap in understanding how soybean populations with contrasting relative maturity patterns respond to environmental variation. We hypothesized that: (i) soybean populations derived from crosses between parents contrasting in relative maturity group would differ in the duration and environmental responsiveness of their phenological periods across growing seasons; (ii) sufficient genetic variability would be present within each population to allow for the identification of lines combining earliness and high grain yield through REML/BLUP; and (iii) temperature and precipitation would significantly influence the duration of the phenological cycle. Accordingly, the objectives of the present study were to: (i) characterize the response of soybean populations in terms of the duration of phenological periods across different growing seasons, considering populations derived from crosses contrasting in RMG; (ii) select lines with high performance for yield and earliness using REML/BLUP within each population; and (iii) evaluate the effects of precipitation and temperature on the phenological cycle of these populations.

## 2. Materials and Methods

### 2.1. Experimental Area Characterization

The experiments were conducted at the Teaching, Research and Extension Farm (FEPE) of FCAV—UNESP, Jaboticabal Campus. The municipality is located in the northwestern region of the state of São Paulo, at latitude 21°14′59″ S and longitude 48°17′8″ W, at an altitude of 575 m. The soil in the area is classified as a eutrophic dark-red Latosol, with a very clayey texture and gently undulating relief. According to the Köppen–Geiger classification, the climate of the region is type Cwa [14].

### 2.2. Genetic Material

Two biparental populations were synthesized from parents differing in relative maturity group. Population E2 was obtained from the cross BMX Potência RR (RMG 6.7) (♀) × BMX Energia RR (RMG 5.3) (♂), corresponding to a parental contrast of 1.4 RMG units. Population E3 was obtained from the cross BRS 245 RR (RMG 7.3) (♀) × BRS 278 RR (RMG 9.4) (♂), corresponding to a parental contrast of 2.1 RMG units [15]. The parents of Population E2 represented early and intermediate maturity cycles, whereas those of Population E3 represented intermediate and late maturity cycles.

### 2.3. Experimental Design and Treatments

The experiments was conducted using an augmented block design [16] with five blocks. The lines were evaluated in unreplicated single-row plots, 5 m long and spaced 0.5 m apart, corresponding to a useful area of 2.5 m^2^. Within each block, the lines were randomly allocated to the experimental plots. Three check cultivars were systematically interspersed throughout the blocks, with each check represented twice per block, totaling 30 check plots across the experiment. At least one of the checks corresponded to one of the parental cultivars.

The checks used were: BMX Potência RR (6.7), AS 3680 IPRO (6.8), and M 6410 (6.4) for Population E2; and BRS 245 RR (7.3), HO APORE IPRO (7.5), and BS 2606 IPRO (6.0) for Population E3. The selection of checks was based on their maturity group proximity to that of the parental lines of the populations.

The populations were evaluated during four growing seasons, and the same set of inbred lines from each population was evaluated in all seasons. Sowing was performed on 23/11/2020 in 2020/2021, 16/11/2021 in 2021/2022, 21/11/2022 in 2022/2023, and 23/11/2023 in 2023/2024. Population E2 (F7:10) consisted of 150 lines, while Population E3 (F7:10) consisted of 80 lines, all at advanced generations of inbreeding.

#### Management of the Experimental Area

Management practices followed the Embrapa recommendations [17]. The experiments were established under a no-tillage system, using a sowing density of 18 plants per meter. Soil fertility was evaluated annually to guide the application of amendments and fertilizers. Seeds were inoculated with bacteria of the genus *Bradyrhizobium*, and pest, disease, and weed management was performed as needed. Irrigation was applied only when water deficit occurred between sowing and crop emergence.

### 2.4. Evaluated Traits

#### 2.4.1. Agronomic Traits

The evaluated traits were the number of days to flowering (NDF), number of reproductive days (NRD), number of days to maturity (NDM), and grain yield (GY). The NDF trait was assessed at the R1 phenological stage when plants transition from the vegetative to the reproductive phase. The NRD trait was calculated as the number of days from R1 to R8, and NDM was assessed at the R8 developmental stage, which corresponds to the period from sowing to the point when at least 50% of the plants have 95% or more mature pods, according to the classification of Fehr and Caviness [18] and is expressed in days. GY was obtained from the grain weight of the plot useful area (2.5 m^2^) after harvest and threshing. The data obtained in grams per plot were converted to kilograms per hectare (kg ha^−1^) and adjusted to 13% moisture content.

#### 2.4.2. Environmental Variables

In addition to the phenotypic evaluation of the traits, environmental variables associated with the lines were also assessed, namely accumulated precipitation (mm) and mean temperature (°C) during the cropping cycle. These variables were calculated individually for each line, considering the duration of each studied phenological period, to represent the climatic conditions experienced by each line throughout its development. This approach was adopted because fixed calendar windows could include periods in which earlier lines had already completed the evaluated phenological stage, potentially misrepresenting their environmental exposure. Nevertheless, considering the potential circularity associated with defining environmental exposure windows based on line-specific phenological information, a sensitivity analysis using fixed post-sowing intervals was also performed. The meteorological data used in this study were provided by the Agroclimatological Station of the Department of Exact Sciences at FCAV/UNESP, Jaboticabal Campus.

### 2.5. Statistical Analyses

To visualize the behavior of NDF, NRD, NDM, and GY across growing seasons, boxplot graphs were used, as they allow for a comparison among cropping years, detection of patterns, and identification of differences related to line performance. Missing observations, when present, were recorded as missing values and were not imputed before statistical analysis.

The linear mixed model used follows the structure presented by Bates et al. [19], defined by the conditional distribution of the response variable Y given the vector of random effects B:

$$\left(Y|B\;=\;b)\;\sim N(X\beta +Zb,\;\sigma^{2}W^{-1}\right)$$

where Xβ represents fixed effects, Zb random effects, $\sigma^{2}$ is the scale parameter (residual variance), and $W^{-1}$ is a diagonal matrix of known weights (assumed to be the identity).

For each experiment, NDM and GY data were fitted using linear mixed models that accounted for the structure of the augmented block design (ABD), with genetic effects treated as random, using the *lme4breeding* package [20]. The model used was as follows:

$$y_{ijk}=\;\mu +\;a_{i}+c+\;g_{j}+\;{\left(g\;x\;gs\right)}_{ij}+b_{k(i)}+\;\varepsilon_{ijk}$$

where:

$y_{ijk}$: observed value (NDM or GY);

μ: overall mean of the trait;

$a_{i}$: fixed effect of growing season i;

c: fixed effect of checks;

$g_{j}$: random effect of genotype j;

${\left(g\;x\;gs\right)}_{ij}$: random effect of genotype x growing season interaction;

$b_{k(i)}$: random effect of block;

$\varepsilon_{ijk}$: experimental error.

The quality of the models was evaluated using the coefficient of determination (R^2^), with a decomposition of these coefficients into fixed and random effects, estimated using the *rsq* package. To assess the significance of fixed effects in the mixed model, a Wald-based ANOVA (Type III) was performed.

Residual diagnostics were performed for all fitted mixed models to assess normality and homogeneity of variance. For grain yield (GY), residuals did not show relevant deviations from normality or homoscedasticity. For number of days to maturity (NDM), some deviation from these assumptions was observed; however, since variance components and fixed-effect estimates were obtained via restricted maximum likelihood (REML) within a linear mixed-model framework, which is comparatively robust to moderate departures from normality and homogeneity of variance, this deviation was not considered to materially compromise the reliability of the genetic parameter estimates or the significance tests reported for this trait.

The variance components ($\sigma_{g}^{2},\;\sigma_{gxgs}^{2},\;\sigma_{e}^{2})$ were estimated by restricted maximum likelihood (REML). The BLUPs (best linear unbiased predictors) were extracted from the estimated random effects in the mixed models. Two-dimensional scatter plots were generated between NDM and GY BLUPs to identify dual-purpose lines for earliness and higher productivity in each population.

The broad-sense heritability $\left(H^{2}\right)$ was estimated from the variance components for each population, considering the different years. According to the equation:

$$H^{2}=\;\frac{\sigma_{g}^{2}}{\sigma_{g}^{2}+\frac{\sigma_{gxgs}^{2}}{a}+\frac{\sigma_{e}^{2}}{ar}}$$

where a is the number of environments, corresponding to the evaluated growing seasons, and r is the number of within-environment replications. In the augmented block design used in this study, test lines were unreplicated within each growing season, therefore, r was considered equal to one for these lines. The replicated checks and blocks contributed to the adjustment of local environmental heterogeneity and to the estimation of experimental error, but they did not constitute replications of the test lines. The selective accuracy of the BLUPs was estimated as $r_{\hat{g}g}=\;\sqrt{H^{2}}$. To assess experimental precision, the experimental coefficient of variation ${(CV}_{e})$ was calculated, as well as the ratio between the genetic coefficient of variation and the experimental coefficient of variation $\left(\frac{{CV}_{g}}{{CV}_{e}}\right)$.

Additionally, mixed models were fitted for NDF, NRD, and NDM, using environmental covariates as fixed effects to evaluate their influence on the phenotypic response of the populations, using the *lme4* package [19]. Preliminary analyses indicated collinearity between accumulated precipitation and mean temperature (|r| > 0.90), so these covariates were fitted in separate single-covariate models to avoid unstable coefficient estimates. The genotype × growing-season interaction were included as random effects, whereas checks were included as a fixed effect. Blocks nested within growing season were also fitted as a random effect, except when the corresponding variance component was estimated as approximately zero. The growing-season main effect was not included separately because the environmental covariates were defined at the growing-season level; therefore, including both terms would confound the effect of the climatic covariate with the effect of growing season. The model was specified in R as response ~ covariate + check + (1|genotype:growing_season) + (1|block):

$$y_{ijk}=\;\mu +\;\beta_{1}x_{i}+c+\;{\left(g\;x\;gs\right)}_{ij}+b_{k(i)}+\;\varepsilon_{ijk}$$

where:

$y_{ijk}$: observed value (NDF, NRD and NDM);

μ: overall mean of the variable;

$\beta_{1}x_{i}$: fixed effect of the environmental covariate $x_{i}$ (accumulated precipitation or mean temperature);

c: fixed effect of checks;

${\left(g\;x\;gs\right)}_{ij}$: random effect of the genotype x growing season interaction;

$b_{k(i)}$: random effect of block;

$\varepsilon_{ijk}$: experimental error.

Marginal prediction plots were generated, allowing for visualization of the effect of environmental covariates on NDF, NRD, and NDM, using the *ggeffects* package. Model quality was evaluated using the coefficient of determination (R^2^), both marginal and conditional, estimated using the performance package, where marginal R^2^ refers to the variance explained only by fixed effects and conditional R^2^ represents the total variance explained by the model. The β coefficients were extracted from the fixed effects estimated by the mixed model. To assess the significance of fixed effects in the mixed model, a Wald-based ANOVA (Type III) was performed. All statistical analyses and plots were performed using R software version 4.4.3 (R Core Team, R Foundation for Statistical Computing, Vienna, Austria) [21], through the RStudio integrated development environment version 2024.12.1.563 (Posit Software, PBC, Boston, MA, USA).

## 3. Results

The distributions of accumulated precipitation (mm) and temperatures (°C) differed among the evaluated growing seasons. The 2020–2021 and 2021–2022 seasons showed similar temperature and precipitation patterns, whereas the 2022–2023 season was the coolest and had the highest rainfall intensity. In contrast, the 2023–2024 season presented the highest temperatures and the lowest precipitation levels (Figure S1).

**Figure 1:**
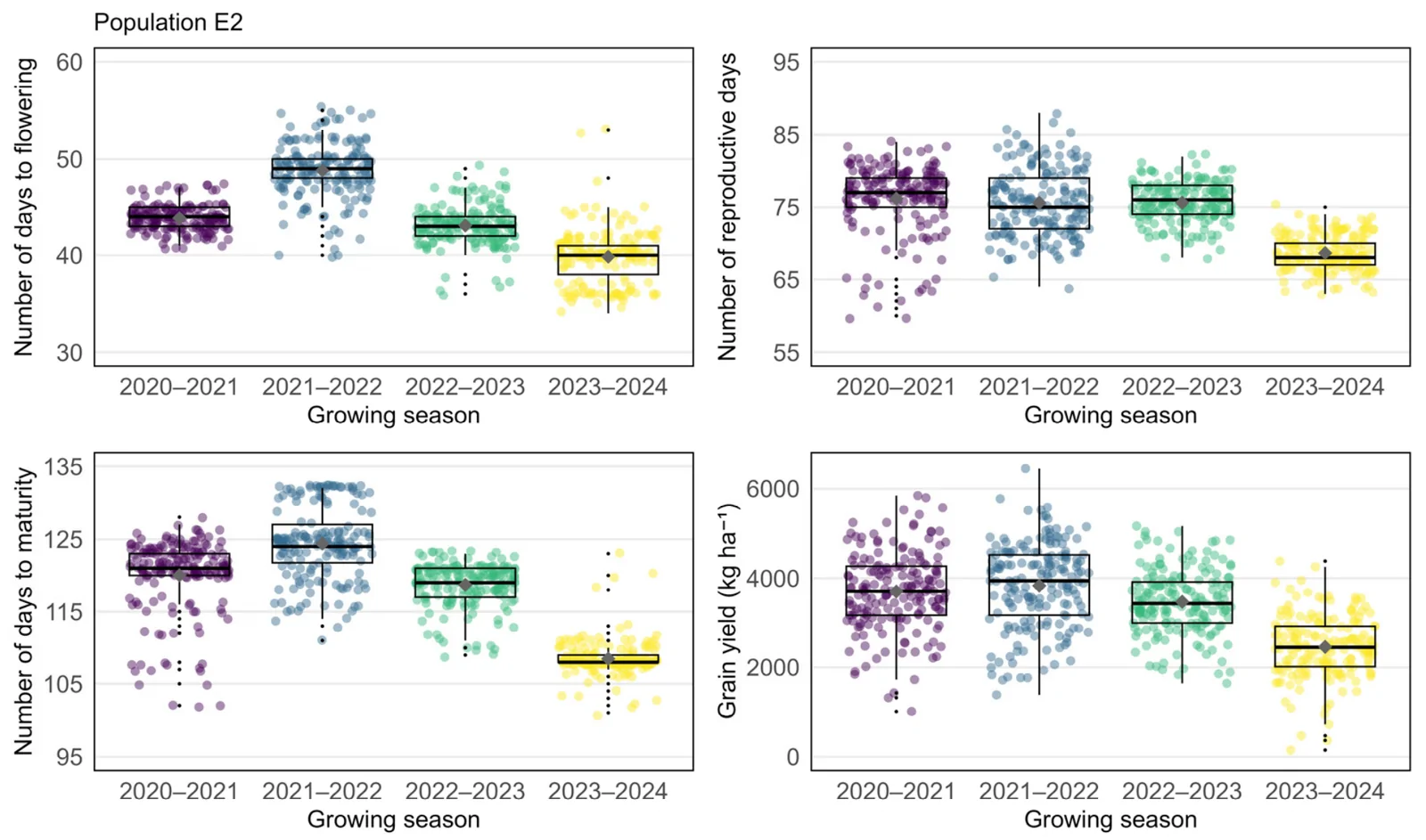
Distribution of the number of days to flowering, number of reproductive days, number of days to maturity, and grain yield for Population E2 across different growing seasons. The boxes indicate the median, quartiles, and overall range of the data, including potential outliers. Each point represents an experimental plot.

The traits NDF, NRD, NDM, and GY of Population E2 exhibited different behaviors across the growing seasons (Figure 1). For NDF in Population E2, similar patterns were observed in the 2020–2021 and 2022–2023 seasons, with similar mean flowering times (43.8 ± 1.3 and 43.1 ± 2.1 days, respectively). In the 2021–2022 season, the plants exhibited delayed flowering (48.8 ± 2.9 days), while in the 2023–2024 season, flowering occurred earlier (39.9 ± 2.9 days). For NRD and NDM in Population E2, similar patterns to NDF were observed across all growing seasons, with the duration of the reproductive period and time to maturity showing consistent behavior across the different years. Regarding GY, a progressive decrease was observed across the growing seasons (Figure 1).

**Figure 2:**
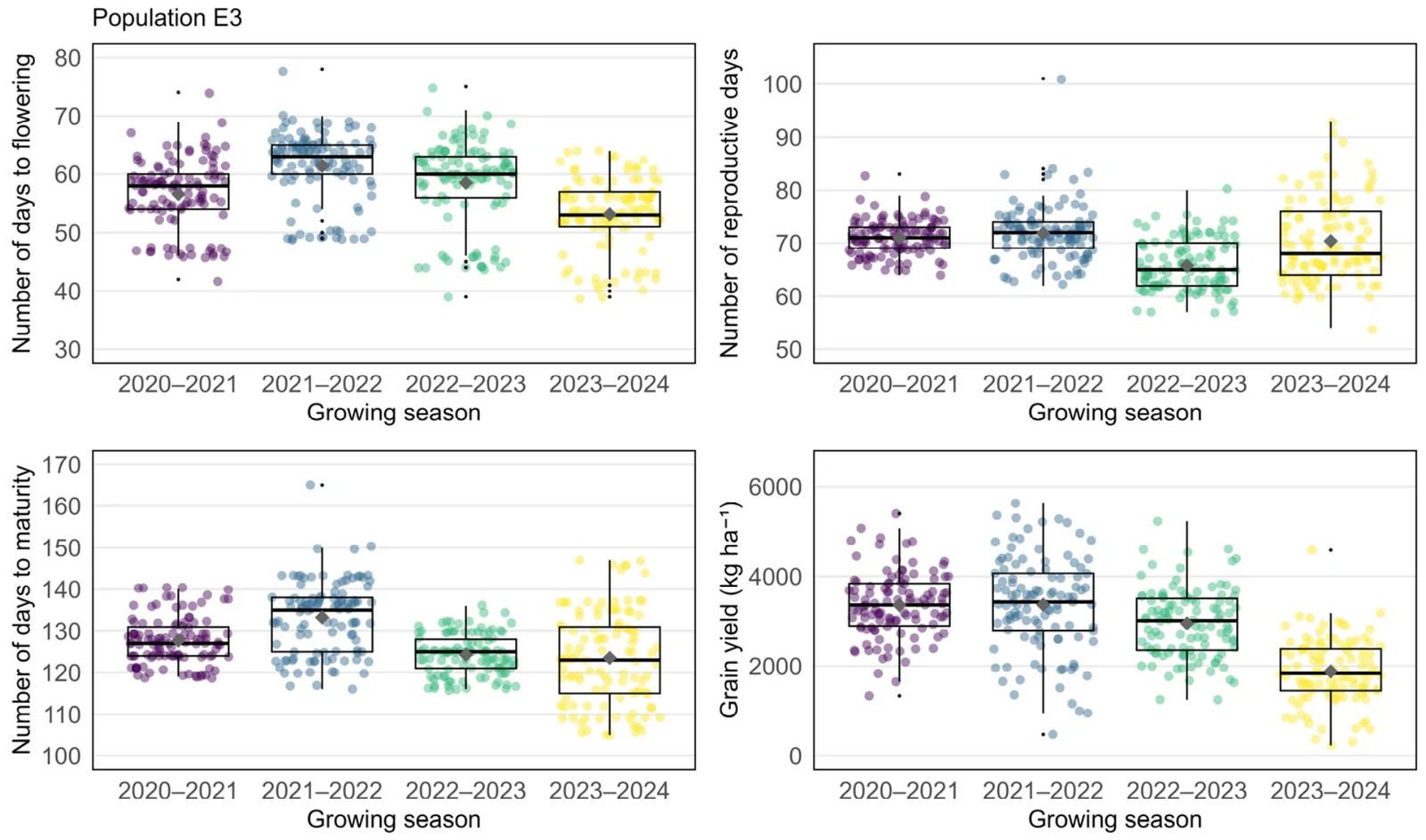
Distribution of the number of days to flowering, number of reproductive days, number of days to maturity, and grain yield for Population E3 across different growing seasons. The boxes indicate the median, quartiles, and overall range of the data, including potential outliers. Each point represents an experimental plot.

Population E3 exhibited later flowering compared to Population E2, and the influence of growing season on this trait was also evident. In the first three seasons, similar NDF patterns were noted (56.6 ± 6.1, 61.4 ± 6.0 and 58.5 ± 7.5 days, respectively), while in the 2023–2024 season, an earlier flowering was observed (53.1 ± 6.2 days) (Figure 2). Regarding NRD, it was observed that in the 2023–2024 season, when flowering occurred earlier, the number of reproductive days of the plants was higher than in previous seasons. The same NRD pattern was observed in NDM. As in Population E2, GY in Population E3 showed a progressive decrease across the seasons (Figure 2). 

For the population’s behavior, the growing season had a significant effect in all evaluated years; in some cases, the checks did not show a significant effect (Table 1). The coefficients of determination of each model were generally high, except for GY, with alternating importance between fixed and random effects across models. For NDF in populations E2 and E3, the variation was mostly accounted for by the fixed effects (0.65 and 0.76, respectively). For NRD, in Population E2, the variation was also primarily due to the fixed effects (0.43), whereas in Population E3, it was mainly attributable to the random effects (0.68). For NDM in populations E2 and E3, the random factors represented the main component of the variation (0.69 and 0.56, respectively). For GY, in the models for populations E2 and E3, the explanatory power was relatively low, but most of the variation was still due to the random effects (0.32 and 0.36, respectively) (Table 1).

In Population E2, the genotypic variance for NDF, NRD, and NDM was higher than the other variance components (2.65, 4.80, and 9.41, respectively), except for GY, which showed a high residual variance. The same pattern was observed in Population E3 for NDF, NRD, and NDM (10.21, 15.84, and 21.23), as well as a high residual variance for GY. The genotype × year interaction variance showed changes depending on the population and the trait analyzed, while the block variance was relatively low (Table 2).

NDF and NDM exhibited high heritabilities, NRD showed intermediate heritability, and GY presented lower heritability, especially in Population E3. The CVg/CVe ratio was greater than 1 for NDF, NRD, and NDM in both populations, indicating potential for selection on these traits, as well as the selective accuracy of the BLUPs. Regarding the means, Population E2 was characterized as earlier than Population E3 for both NDF and NDM. For NRD, Population E2 had a higher mean than Population E3. Concerning GY, Population E2 (3553.15 kg ha^−1^) showed better performance than Population E3 (2953.71 kg ha^−1^) (Table 2).

**Figure 3:**
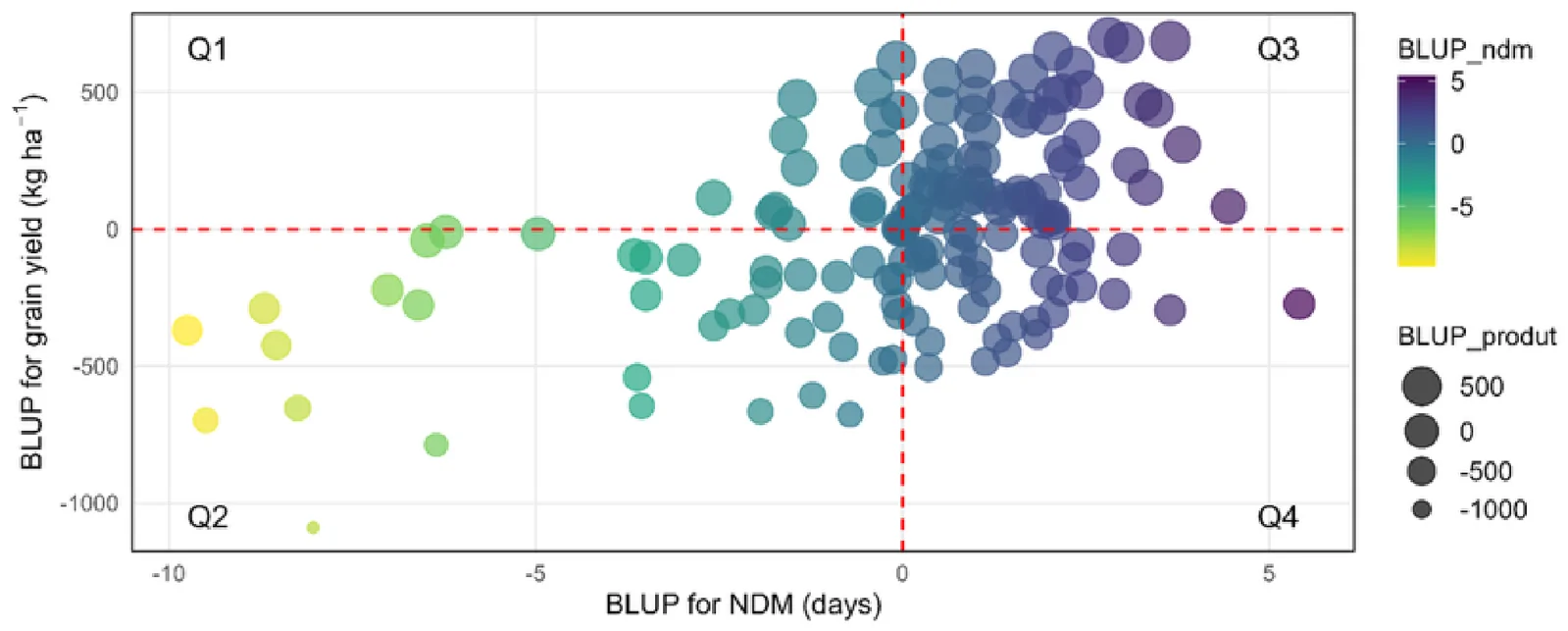
Two-dimensional dispersion of the BLUPs for number of days to maturity (NDM) and grain yield (GY) of Population E2. The red dotted lines define the four performance quadrants relative to the population mean: Q1 (below–average NDM and above–average yield), Q2 (below–average NDM and below–average yield), Q3 (above–average NDM and above–average yield), and Q4 (above–average NDM and below–average yield).

**Figure 4:**
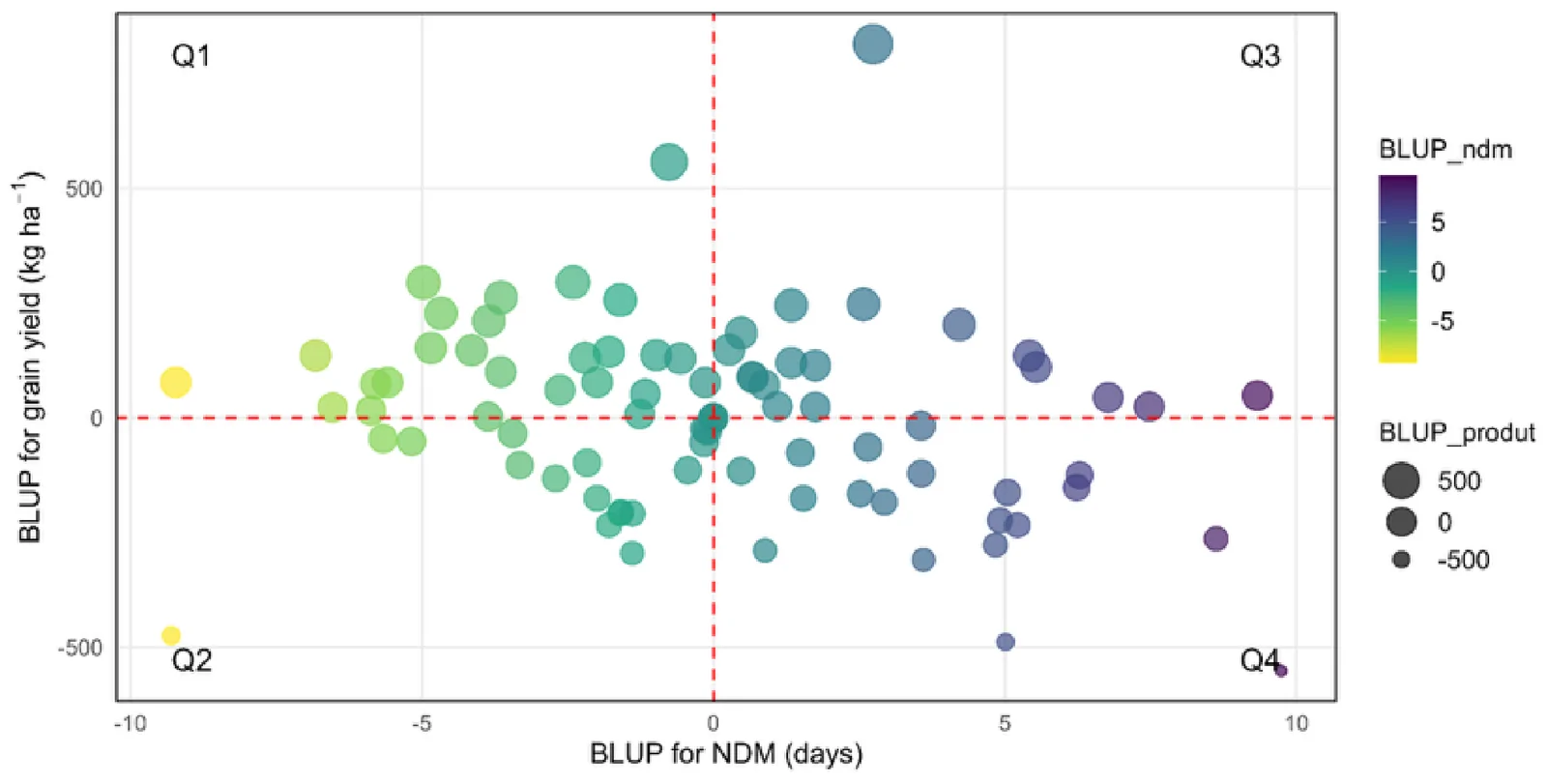
Two-dimensional dispersion of the BLUPs for number of days to maturity (NDM) and grain yield (GY) of Population E3. The red dotted lines define the four performance quadrants relative to the population mean: Q1 (below–average NDM and above–average yield), Q2 (below–average NDM and below–average yield), Q3 (above–average NDM and above–average yield), and Q4 (above–average NDM and below–average yield).

Figure 3 and Figure 4 show a dispersion of the BLUPs estimated for NDM and GY. The zero (0) point on the axes represents the overall mean for each trait. In this context, positive BLUPs for NDM indicate late lines, while negative BLUPs indicate early lines, based on the population mean. The same applies to GY, where lines with positive BLUPs are more productive and lines with negative BLUPs are less productive.

In Population E2 (Figure 3), BLUPs for NDM ranged from –10 to +5 days, representing a difference of 15 days between the earliest and latest lines. For GY, BLUPs ranged from –1089 to +700 kg ha^−1^. A complete list of inbreed lines with their BLUP values for NDM and GY is provided in the Supplementary Materials (Table S1).

For Population E3 (Figure 4), BLUPs for NDM ranged from –9 to +9 days, representing a difference of 18 days between the earliest and latest lines in the cycle. For GY, BLUPs ranged from –550 to +814 kg ha^−1^. A full list of inbred lines is provided in the Supplementary Materials (Table S2).

The five most promising lines, combining below-average NDM and above-average grain yield relative to their respective population means, were POEN-58-108.1, POEN-114-108.2, POEN-35-147.3, POEN-64-31.1, and POEN-81-183.1 in Population E2, and BR245BR278-5-42.1, BR245BR278-55-3.2, BR245BR278-34-68.5, BR245BR278-45-3.1, and BR245BR278-48-68.3 in Population E3. Their predicted NDM and grain yield values are presented in Table 3.

**Figure 5:**
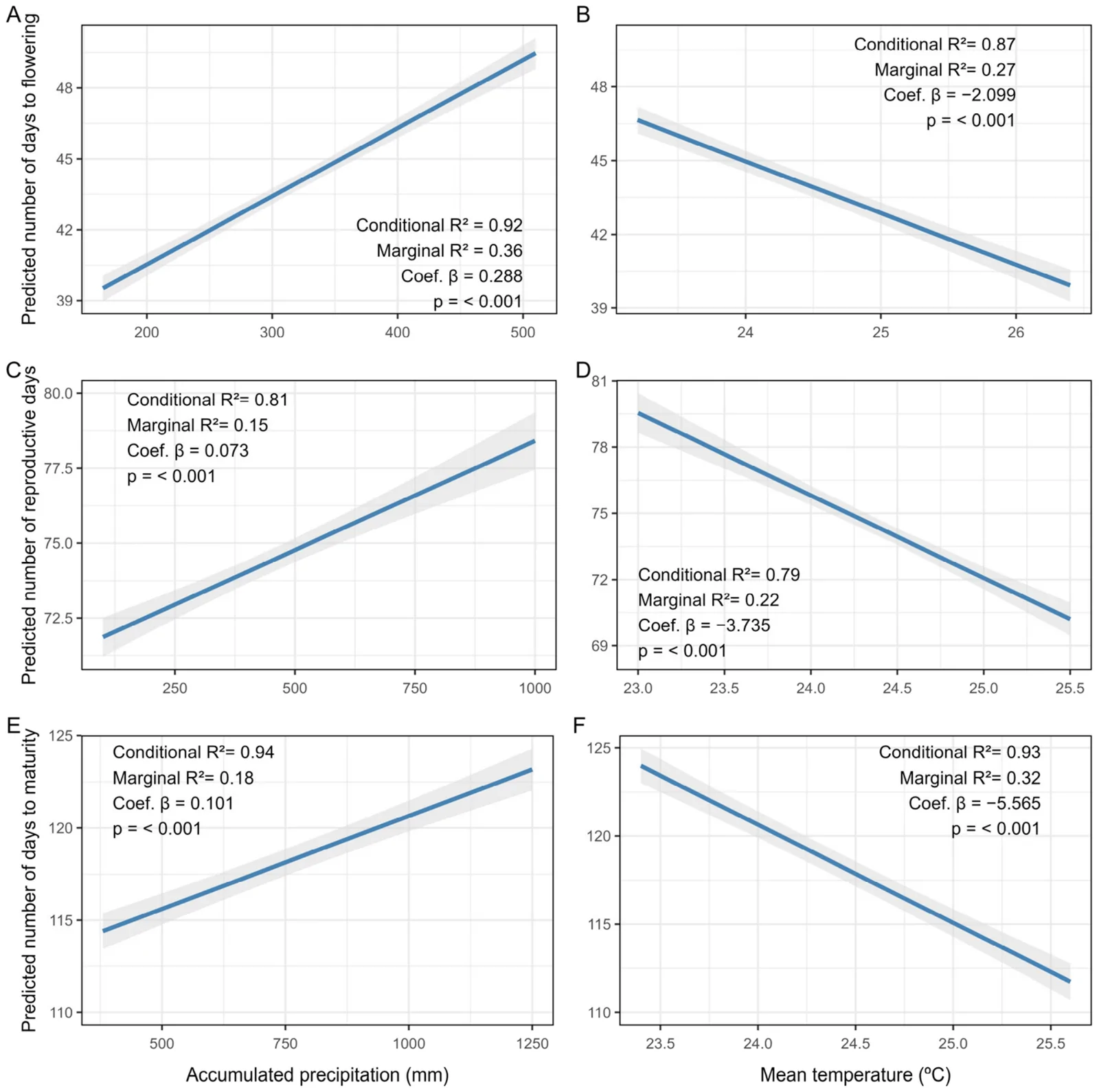
Marginal predictions of NDF, NRD, and NDM obtained from mixed models adjusted with accumulated precipitation and mean temperature as fixed covariates for Population E2: (**A**) NDF as a function of accumulated precipitation; (**B**) NDF as a function of mean temperature; (**C**) NRD as a function of accumulated precipitation; (**D**) NRD as a function of mean temperature; (**E**) NDM as a function of accumulated precipitation; and (**F**) NDM as a function of mean temperature. Blue lines represent the model-adjusted predictions, and the shaded areas represent the corresponding 95% confidence intervals. The fitted relationships represent statistical associations and should not be interpreted as evidence of causal effects. Jaboticabal, SP.

**Figure 6:**
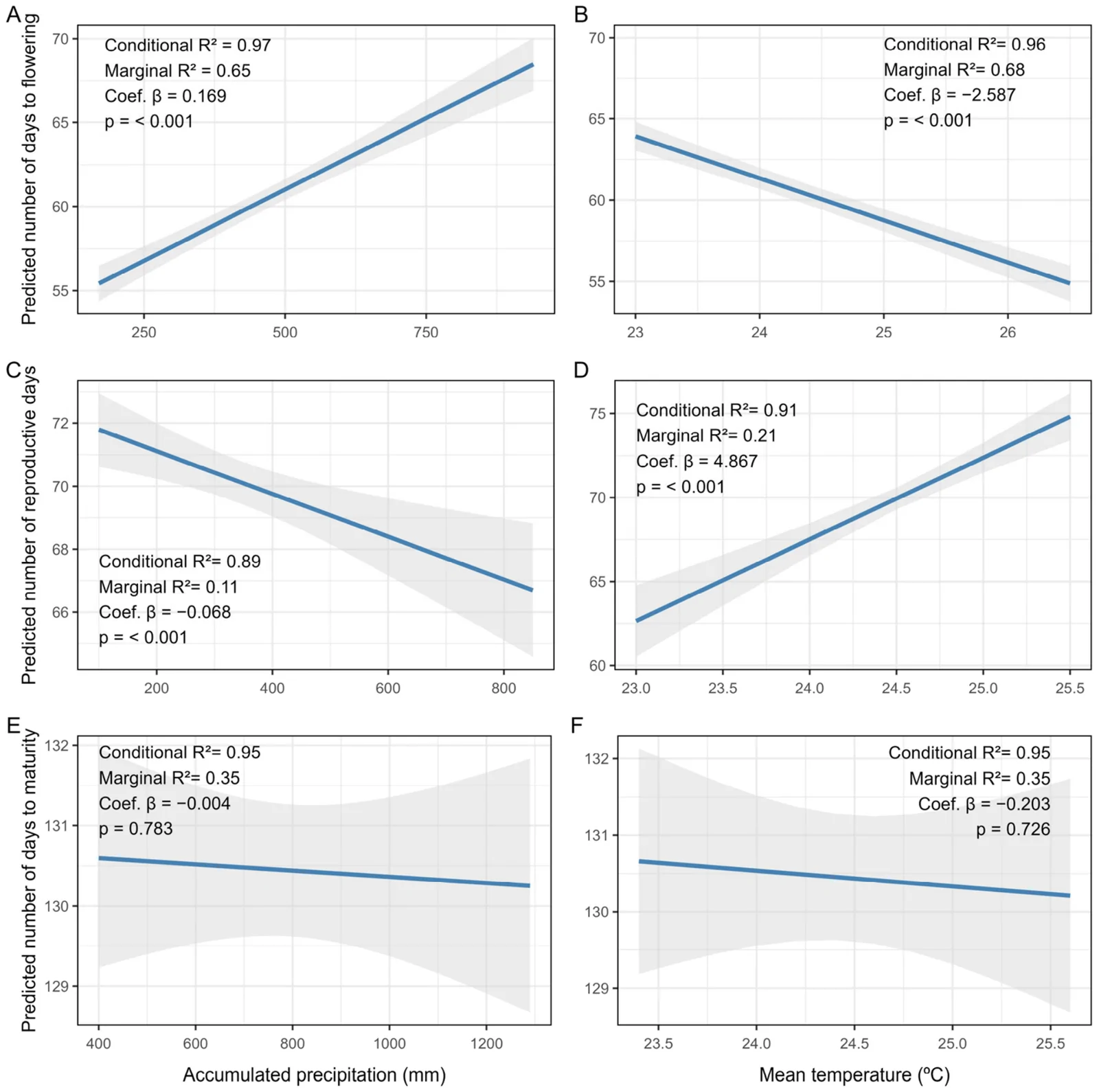
Marginal predictions of NDF, NRD, and NDM obtained from mixed models adjusted with accumulated precipitation and mean temperature as fixed covariates for Population E3: (**A**) NDF as a function of accumulated precipitation; (**B**) NDF as a function of mean temperature; (**C**) NRD as a function of accumulated precipitation; (**D**) NRD as a function of mean temperature; (**E**) NDM as a function of accumulated precipitation; and (**F**) NDM as a function of mean temperature. Blue lines represent the model-adjusted predictions, and the shaded areas represent the corresponding 95% confidence intervals. The fitted relationships represent statistical associations and should not be interpreted as evidence of causal effects. Jaboticabal, SP.

To visualize the influence of the environment on the variation of NDF, NRD, and NDM, mixed models were fitted for each population using the environmental covariates accumulated precipitation and mean temperature. The models exhibited high explanatory power, with conditional R^2^ ranging from 0.79 to 0.97 (Figure 5 and Figure 6).

The environmental covariates were significant (*p* < 0.001) in all models fitted for Population E2 (Table S3). Lower precipitation levels were associated with earlier flowering, while increased precipitation was associated with later flowering. The model exhibited high explanatory power (R^2^ = 0.92), with a considerable contribution from accumulated precipitation (R^2^ = 0.36), presenting a positive β coefficient, that is, for each 10 mm increase in precipitation, NDF increased by 0.28 days (Figure 5A, Table S3). The estimated β coefficients, standard errors, and 95% confidence intervals are presented in the Supplementary Materials (Table S3).

Lower temperatures were associated with later flowering, while higher temperatures were associated with earlier flowering. The model showed an explanatory power of 0.87, with mean temperature as a fixed effect explaining 0.27. The β coefficient was negative; that is, for each 1 °C increase in mean temperature, NDF decreased by 2.09 days (Figure 5B, Table S3).

Regarding NRD, the pattern was repeated: increased precipitation was associated with a higher number of reproductive days, while increased mean temperature was associated with a reduction in NRD. The models showed explanatory powers of 0.81 and 0.79, respectively, and a low contribution from the fixed effects (0.15 and 0.22, respectively), with a β coefficient of 0.073 for accumulated precipitation and −3.73 for mean temperature (Figure 5C,D, Table S3).

For NDM, that is, considering the entire cycle of the lines, the environmental covariates had significant effects, although their contributions were smaller than those of the random effects, with R^2^ values of 0.18 for accumulated precipitation and 0.32 for mean temperature. The full models exhibited high explanatory power (0.94 and 0.93, respectively). For NDM, each 10 mm increase in precipitation was associated with an increase of 0.101 days in the total cycle, while each 1 °C increase was associated with a reduction of 5.56 days in the total cycle of the lines (Figure 5E,F, Table S3).

In Population E3, for NDF, increased precipitation was associated with later flowering of the lines, whereas higher temperature was associated with earlier flowering of the population. The models showed good fit, with a conditional R^2^ of 0.97 for accumulated precipitation and 0.96 for mean temperature, such that the fixed effects (0.65 and 0.68, respectively) were highly important in explaining the variation in flowering. The β coefficient was 0.169 for accumulated precipitation and −2.587 for mean temperature (Figure 6A,B, Table S3).

In contrast to what was observed for Population E2, regarding NRD, in Population E3, increased precipitation was associated with a shortening of the reproductive days, while increased temperature was associated with a prolongation of these days. The models showed high R^2^ values (0.89 and 0.91, respectively) but with a low contribution from the fixed effects (0.11 and 0.21, respectively). The β coefficient was negative for precipitation (−0.068) and positive for mean temperature (4.867) (Figure 6C,D, Table S3).

For NDM, both models showed a conditional R^2^ of 0.95. However, the fixed effects were not significant (*p* = 0.783 for accumulated precipitation and *p* = 0.726 for mean temperature), showing no significant association with variation in the total cycle duration of the lines (Figure 6E,F, Table S3). The decomposition of the coefficients of determination into fixed and random effects is available in the Supplementary Materials (Table S4).

Overall, the patterns obtained with fixed windows were consistent with those identified using line-specific phenological intervals, supporting the robustness of the environmental associations observed. However, some differences were detected for NRD in Population E3, where the associations with precipitation and temperature differed according to the environmental window definition (Figures S2 and S3, Table S5).

## 4. Discussion

The variability observed (Figure 1 and Figure 2) for NDF, NRD, NDM, and GY in the populations can be attributed to a combination of genetic and environmental factors: genetic factors associated with the maturity group (RMG) of each line, and consequently, adaptation to the location where the experiments were conducted; and environmental factors related to the climatic conditions of each growing season (Figure S1). Population E2, derived from parents differing by 1.4 RMG units, showed lower genotypic variance for NDF, NRD, and NDM and was characterized by earlier maturity. In contrast, Population E3, derived from parents differing by 2.1 RMG units, showed higher genotypic variance for these phenological traits (Table 2). The greater parental contrast in RMG may have contributed to the broader phenological variation observed in E3; however, genetic distance between the parents was not directly assessed in this study. Jean et al. [22] reported that greater parental divergence can increase the variability expressed among progenies, whereas crosses between more similar parents may produce narrower variation for the trait under evaluation.

Regarding the check cultivars, a significant effect (*p* < 0.001) was detected for NDF and NDM in Population E3 (Table 1), whereas no significant check effect was observed in Population E2 for any trait. This result likely reflects genetic differences among the three check cultivars used in Population E3 (BRS 245 RR, HO APORE IPRO, and BS 2606 IPRO), which covered a broader range of relative maturity groups than those used in Population E2. The significant check effect therefore indicates that these cultivars captured meaningful differences in phenological behavior and supported the adjustment of the unreplicated lines.

The growing seasons strongly influenced the phenological traits and were also associated with variation in GY, as the same genotypes showed different performances across seasons (Figure 1 and Figure 2 and Figure S1, Table 1). However, the progressive reduction in GY should be interpreted cautiously because, in addition to seasonal environmental variation, the experimental design may have contributed to unexplained variation. Gong et al. [23] reported that variation in precipitation, temperature, and sunlight duration can shorten or extend the soybean phenological cycle. These responses reflect genotype × environment interactions. Under stress conditions, such as reduced precipitation and increased temperature, plants may show shorter developmental periods and reductions in yield-related traits [24].

High broad-sense heritability estimates for NDF, ranging from 0.77 to 0.90 in both populations, demonstrate the strong genetic control of this trait, as also reported in the literature: 0.82 [25], 0.88 [26], and 0.94 [27]. In addition, flowering has a strong genetic component, although environmental heterogeneity is a crucial factor contributing to variation in flowering time in soybean [28], as also observed in our results. NRD in both populations showed intermediate heritability (0.68 and 0.66) and higher variance compared to NDF, indicating that environmental conditions influenced the duration of the reproductive phase, and consequently, the relative allocation of developmental time between the vegetative and reproductive phases [29]. NDM (Table 2), ranging from 0.82 to 0.84, showed that despite environmental influence on the crop cycle, the genetic component plays a predominant role in the phenotypic expression of the trait, a fact widely reported in the literature. Previous studies have reported similar or higher heritability values, with estimates of 0.72 [26], 0.85 [30], 0.91 [31], 0.93 [32], and up to 0.94 [27].

For GY, the largest variance component was the residual, indicating that this trait was strongly influenced by environmental variation [33] and by factors not fully captured by the model. In addition, the use of single, unreplicated rows without border rows may have increased experimental noise through inter-plot competition and border effects, contributing to the high residual variance and lower heritability estimates. Heritability for GY was lower than that observed for the phenological traits, ranging from 0.43 to 0.62 across the two populations (Table 2). Therefore, the genetic and environmental interpretation of grain yield should be made cautiously. These estimates are consistent with values reported in previous studies, including 0.59 [26] and 0.62 [30].

The use of mixed models through BLUPs in several breeding programs has proven to be an efficient tool for the prediction of genetic values and selection support, especially for quantitative traits influenced by the environment [34,35,36,37]. The genetic variability observed within each population allows for the identification and selection of lines with superior performance for earliness and grain yield, as also reported by Bianchi et al. [27]. Based on predicted genetic values, the BLUP approach supports the simultaneous selection of lines combining shorter maturity cycles and high productivity. Thus, this methodology provides an effective strategy for the integrated selection of dual-purpose genotypes based on phenological and productive traits [38].

The contrasting phenological responses observed between Populations E2 and E3 may reflect differences in photoperiod sensitivity, thermal requirements, and the genetic background controlling flowering and maturity. Soybean phenology is regulated by interconnected photoperiodic and circadian pathways involving maturity genes and FT-related genes [7,8,9]. Although these mechanisms were not directly evaluated in the present study, they provide a plausible biological framework for interpreting the population-specific variation in NDF, NRD, and NDM.

In Population E2 (Figure 5A–F), accumulated precipitation was positively associated with the duration of the phenological stages, whereas mean temperature showed a negative association in both growing periods. This pattern suggests that greater water availability may prolong developmental processes, while higher temperatures increase developmental rates and shorten the crop cycle. Conversely, reduced precipitation may intensify water limitation and contribute to the earlier completion of developmental stages. These responses indicate that the phenological cycle of Population E2 was influenced by variation in water availability and thermal conditions, consistent with previous reports [10,24,39,40].

Liu and Dai [39], when evaluating the phenological response of soybean under different precipitation and temperature conditions, observed distinct effects depending on the developmental stage. In the vegetative period, variations of −0.01 to −0.11 days/10 mm of precipitation and −0.63 to −2.51 days/°C of mean temperature were reported. In the reproductive period, the effects ranged from −0.02 to 0.11 days/10 mm and −0.98 to 0.56 days/°C, indicating greater variability in the climatic response during this phase. For the total crop cycle, precipitation showed effects ranging from −0.06 to 0.09 days/10 mm, while temperature reduced the cycle duration by approximately 2.51 to 5.19 days/°C, depending on the region and growing season. For Population E2, precipitation showed positive β coefficients for flowering (0.42 days/10 mm), the reproductive period (0.052 days/10 mm), and maturity (0.101 days/10 mm), indicating a slight extension of these stages. Conversely, mean temperature showed negative coefficients for flowering (−2.289 days/°C), the reproductive period (−3.502 days/°C), and maturity (−5.565 days/°C), indicating progressively shorter phenological stages with increasing temperature (Figure 5A–F).

For Population E3, the same pattern observed in Population E2 for NDF was maintained regarding the effects of precipitation and temperature. For NRD, a shortening of the period was observed with increasing precipitation, and an extension of the period with increasing temperature, although the contribution of these variables was low (R^2^ = 0.11 and 0.21, respectively; Figure 6C,D). This pattern may reflect a hypothetical self-compensation mechanism between phenological phases within the genotype; however, since no physiological measurements (e.g., photoassimilate partitioning or developmental rate at the sub-phase level) were taken in this study, this interpretation remains a hypothesis and warrants direct investigation in future research. For NDM, environmental covariates did not show a significant effect, suggesting that later-maturing genotypes are less sensitive to precipitation and temperature effects, maintaining a more stable phenological pattern across years (Figure 6A–F). This result agrees with Sobko et al. [12], who reported greater environmental sensitivity in early-maturing genotypes, and is also consistent with Xin et al. [41], who showed that soybean phenology responds to climatic variation, particularly temperature and precipitation.

These results indicate that the inclusion of environmental variables in models becomes important for understanding how certain factors influence agronomic traits, allowing for the quantification of specific effects on plant development. However, careful attention must be given to the selection of variables, as they should have a causal or physiological relationship with the process being modeled, and additionally, environmental variables are generally collinear, which can make it difficult to distinguish the true contributions of different climatic factors [10].

Although this study provides relevant evidence on the effect of the environment on the phenological and productive modulation of biparental populations with contrasting maturity patterns, some limitations should be acknowledged. First, the experiments were conducted at a single location, although over four growing seasons. Therefore, although seasonal climatic variation was captured, the extrapolation of the results to other soybean-producing regions with different latitude, soil, altitude, and management conditions should be made with caution. Second, the study was based on only two biparental populations, which limits the generalization of the observed responses to the broader genetic diversity of soybean regarding relative maturity groups. Third, considering the large number of lines evaluated, the use of an augmented block design was an appropriate choice; however, this design did not allow for replication of the lines within each growing season, which could have provided greater experimental precision.

An additional limitation concerns the plot structure used for grain yield estimation. Test lines were evaluated in single, unreplicated 5-m rows without border rows. Although this design is generally suitable for phenological traits, grain yield is more susceptible to inter-plot competition and border effects. Differences in plant architecture, maturity, shading, and resource use among adjacent genotypes may therefore have increased residual variance and contributed to the lower heritability observed for GY (Table 2). Thus, part of the unexplained variation may reflect methodological noise rather than climatic effects alone. Future yield evaluations should consider replicated plots, border rows, or spatial correction methods to improve the separation of genetic and environmental effects.

In addition, the environmental-covariate models focused only on accumulated precipitation and mean temperature, although other factors, such as solar radiation, soil water availability, growing degree days, and biotic stresses, may also influence population responses. Future studies including additional locations, greater genotype diversity across a broader range of RMG, and replicated experimental designs would help confirm the applicability of these findings.

## 5. Conclusions

Population E2 showed lower variability among lines and a predominance of early-maturing genotypes, indicating greater uniformity. In contrast, Population E3 exhibited greater variability and a predominance of late-maturing lines, likely reflecting the greater contrast between its parents in terms of the relative maturity group.

The REML/BLUP approach enabled the estimation of genetic values and the identification of promising lines combining earlier maturity and higher grain yield within each population, confirming the presence of exploitable genetic variability.

Accumulated precipitation and mean temperature were associated with phenological duration in both populations, but the magnitude and direction of these associations differed between them. Population E2 showed more consistent associations between the environmental covariates and NDF, NRD, and NDM, whereas Population E3 showed weaker or nonsignificant associations for some traits, particularly NDM. These results indicate population-specific environmental responses under the conditions evaluated.

## Figures and Tables

**Table 1:** Coefficients of determination (R^2^) and Type III Wald test results for fixed effects in mixed models fitted for NDF, NRD, NDM, and GY in two soybean populations. Jaboticabal, SP.

Population	Trait	Model R^2^	Fixed R^2^	Random R^2^	Fixed Effect	χ^2^	Df	*p*-Value
E2	NDF	0.86	0.65	0.22	Growing season	366.26	3	<0.001 ***
					Check	0.27	2	0.97 ns
	NRD	0.82	0.43	0.39	Growing season	127.39	3	<0.001 ***
					Check	2.98	2	0.40 ns
	NDM	0.93	0.25	0.69	Growing season	453.93	3	<0.001 ***
					Check	1.68	2	0.64 ns
	GY	0.57	0.24	0.32	Growing season	117.52	3	<0.001 ***
					Check	1.95	2	0.58 ns
E3	NDF	0.96	0.76	0.20	Growing season	269.30	3	<0.001 ***
					Check	37.49	2	<0.001 ***
	NRD	0.89	0.21	0.68	Growing season	130.14	3	<0.001 ***
					Check	2.50	2	0.47 ns
	NDM	0.96	0.40	0.56	Growing season	149.40	3	<0.001 ***
					Check	17.30	2	<0.001 ***
	GY	0.54	0.18	0.36	Growing season	111.68	3	<0.001 ***
					Check	0.84	2	0.84 ns

Model R^2^: represents the proportion of variation explained by the complete model, including fixed and random effects; Fixed R^2^: represents the proportion explained exclusively by the fixed effects; Random R^2^: represents the proportion explained exclusively by the random effects. χ^2^: Chi-square; *p*-value: significance of fixed effects in each experiment; Check: check genotype. Significance codes: *** *p*-Value ≤ 0.001; ns: not significant (*p*-Value > 0.1).

**Table 2:** Variance components and genetic parameters for NDF, NRD, NDM, and GY in two soybean populations. Jaboticabal, SP.

Parameter	Population E2				Population E3			
	NDF Days^2^	NRD Days^2^	NDM Days^2^	GY (kg·ha^−1^)^2^	NDF Days^2^	NRD Days^2^	NDM Days^2^	GY (kg·ha^−1^)^2^
	Variance components							
$\sigma_{g}^{2}$	2.65	4.80	9.41	192,826.96	10.21	15.84	21.23	108,691.44
$\sigma_{gxgs}^{2}$	0.95	4.54	3.78	35,436.18	2.27	9.75	14.95	68,051.73
$\sigma_{b(gs)}^{2}$	0.44	1.36	1.35	35,216.48	0.45	0.42	1.10	40,124.40
$\sigma_{e}^{2}$	2.28	4.49	3.36	433,279.93	2.11	3.80	3.18	512,606.14
	Genetic values							
$H^{2}$	0.77	0.68	0.84	0.62	0.90	0.66	0.82	0.43
$r_{\hat{g}g}$	0.88	0.82	0.92	0.79	0.95	0.82	0.91	0.65
${CV}_{e}(\%)$	3.42	2.91	1.56	18.52	2.77	2.82	1.47	24.23
${CV}_{g}(\%)$	3.69	3.00	2.62	12.36	6.10	4.52	3.79	11.16
${{CV}_{g}/CV}_{e}\;ratio$	1.08	1.03	1.67	0.67	2.20	1.60	2.58	0.46
Mean	44.12	72.89	117.01	3553.15	52.38	69.06	121.43	2953.71

$\sigma_{g}^{2}$: genotypic variance; $\sigma_{gxgs}^{2}$: genotype × growing season interaction variance; $\sigma_{b(gs)}^{2}$: variance of blocks within growing seasons; $\sigma_{e}^{2}$: residual variance; $H^{2}$: broad-sense heritability; $r_{\hat{g}g}$: selective accuracy of BLUPs; ${CV}_{e}$: experimental coefficient of variation; ${CV}_{g}$: genotypic coefficient of variation.

**Table 3:** Most promising soybean inbred lines selected for below-average number of days to maturity and above-average grain yield in Populations E2 and E3. Jaboticabal, SP.

Population	Inbred Line	Predicted NDM (Days)	Predicted GY (kg ha ^−1^)
E2	POEN-58-108.1	116.93	4168.04
	POEN-114-108.2	116.62	4069.22
	POEN-35-147.3	115.57	4029.54
	POEN-64-31.1	116.97	3987.98
	POEN-81-183.1	116.75	3959.34
E3	BR245BR278-5-42.1	120.67	3511.39
	BR245BR278-55-3.2	119.02	3249.22
	BR245BR278-34-68.5	116.46	3248.33
	BR245BR278-45-3.1	117.79	3216.28
	BR245BR278-48-68.3	119.83	3210.71

Predicted NDM and GY values were calculated as $\hat{Y_{i}\;}=\;\hat{\mu }+\;{\hat{g}}_{i}$ where $\hat{\mu }$ is the overall model-adjusted mean for the respective population and trait, and ${\hat{g}}_{i}$ is the BLUP of the ith line. Lines were selected from quadrant Q1, characterized by below-average NDM and above-average GY, and ranked according to predicted grain yield.

## Data Availability

The data that support the findings of this study are available from the corresponding author upon request.

## Supplementary Materials

The following supporting information can be downloaded at: https://www.mdpi.com/article/10.3390/biology15171464/s1, Figure S1: Climogram of monthly accumulated precipitation (mm) and mean temperature (°C) during the evaluated growing seasons; Figure S2: Marginal predictions of NDF, NRD, and NDM obtained from mixed models adjusted with accumulated precipitation and mean temperature calculated using fixed post-sowing environmental windows as fixed covariates for Population E2: (A) NDF as a function of accumulated precipitation; (B) NDF as a function of mean temperature; (C) NRD as a function of accumulated precipitation; (D) NRD as a function of mean temperature; (E) NDM as a function of accumulated precipitation; and (F) NDM as a function of mean temperature. Blue lines represent the model-adjusted predictions, and the shaded areas represent the corresponding 95% confidence intervals. The fitted relationships represent statistical associations and should not be interpreted as evidence of causal effects. Jaboticabal, SP; Figure S3: Marginal predictions of NDF, NRD, and NDM obtained from mixed models adjusted with accumulated precipitation and mean temperature calculated using fixed post-sowing environmental windows as fixed covariates for Population E3: (A) NDF as a function of accumulated precipitation; (B) NDF as a function of mean temperature; (C) NRD as a function of accumulated precipitation; (D) NRD as a function of mean temperature; (E) NDM as a function of accumulated precipitation; and (F) NDM as a function of mean temperature. Blue lines represent the model-adjusted predictions, and the shaded areas represent the corresponding 95% confidence intervals. The fitted relationships represent statistical associations and should not be interpreted as evidence of causal effects. Jaboticabal, SP; Table S1: Distribution of inbred lines according to BLUPs for number of days to maturity (NDM) and grain yield (GY) across four performance quadrants (Q1–Q4) for Population E2. Quadrants represent different agronomic profiles relative to the population mean: Q1 (below–average NDM and above–average yield), Q2 (below–average NDM and below–average yield), Q3 (above–average NDM and above–average yield), and Q4 (above–average NDM and below–average yield); Table S2: Distribution of inbred lines according to BLUPs for number of days to maturity (NDM) and grain yield (GY) across four performance quadrants (Q1–Q4) for Population E3. Quadrants represent different agronomic profiles relative to the population mean: Q1 (below–average NDM and above–average yield), Q2 (below–average NDM and below–average yield), Q3 (above–average NDM and above–average yield), and Q4 (above–average NDM and below–average yield); Table S3: Coefficients of determination (R^2^) and results of the Type III Wald test for fixed effects in the mixed models fitted for NDF, NRD, and NDM in two soybean populations, considering two environmental covariates: accumulated precipitation and mean temperature. Jaboticabal, SP; Table S4: Decomposition of the coefficient of determination into fixed and random effects in mixed models fitted for phenological traits in two soybean populations. Jaboticabal, SP; Table S5: Coefficients of determination (R^2^) and results of the Type III Wald test for fixed effects in mixed models fitted for NDF, NRD, and NDM in two soybean populations using accumulated precipitation and mean temperature calculated from fixed post-sowing environmental windows as covariates. Jaboticabal, SP.

## Abbreviations

The following abbreviations are used in this manuscript:

NDF	Number of days to flowering
NRD	Number of reproductive days
NDM	Number of days to maturity
GY	Grain yield
RMG	Relative maturity group
REML	Restricted maximum likelihood
BLUP	Best linear unbiased predictor
