# Supplementary material for: Phenological Development, Environmental Effects, and Selection of Soybean Lines from Crosses with Contrasting Relative Maturity Groups
Source: Biology (Basel). 2026 Aug 28;15(17):1464. doi: 10.3390/biology15171464 (PMC13565565; doi:10.3390/biology15171464)
Supplement: Supplementary file 1 [file biology-15-01464-s001.zip › biology-4456594-supplementary.pdf]

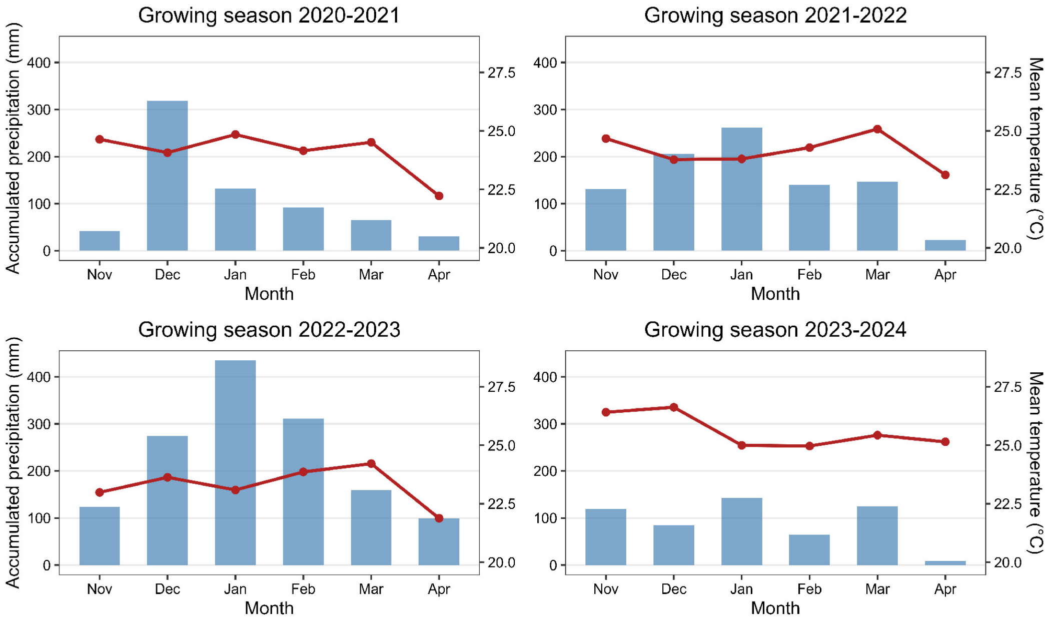

Figure S1. Climogram of monthly accumulated precipitation (mm) and mean temperature (°C) during the evaluated growing seasons.

Table S1. Distribution of inbred lines according to BLUPs for number of days to maturity (NDM) and grain yield (GY) across four performance quadrants (Q1–Q4) for Population E2. Quadrants represent different agronomic profiles relative to the population mean: Q1 (below–average NDM and above–average yield), Q2 (below–average NDM and below–average yield), Q3 (above–average NDM and above–average yield), and Q4 (above–average NDM and below–average yield).

Quadrant	Agronomic class	Inbred line	BLUP NDM	BLUP yield
Q1	Below-average NDM and above-average yield	POEN-58-108.1	-0.08	614.89
		POEN-114-108.2	-0.39	516.07
		POEN-35-147.3	-1.44	476.39
		POEN-64-31.1	-0.04	434.83
		POEN-81-183.1	-0.26	406.19
		POEN-75-62.4	-1.56	342.67
		POEN-95-166.2	-0.25	296.41
		POEN-113-189.4	-0.60	242.40
		POEN-18-114.4	-1.41	224.09
		POEN-47-185.1	-2.57	114.11
		POEN-63-116.1	-0.46	89.18
		POEN-8-191.3	-1.73	71.01
		POEN-60-183.4	-0.47	68.26
		POEN-61-39.4	-1.77	59.61
		POEN-6-192.2	-1.56	18.93
		POEN-20-28.1	-0.05	6.34
Q2	Below-average NDM and below-average yield	POEN-100-78.4	-0.04	-0.80
		POEN-23-191.2	-6.23	-10.40
		POEN-12-182.2	-4.97	-17.20
		POEN-13-70.3	-6.48	-41.76
		POEN-68-171.1	-3.66	-95.42

		POEN-46-120.1	-3.50	-103.85
		POEN-57-126.5	-2.99	-112.58
		POEN-77-40.3	-0.47	-121.00
		POEN-49-138.5	-1.86	-155.17
		POEN-27-192.4	-1.39	-167.11
		POEN-25-45.1	-0.88	-173.58
		POEN-48-45.3	-0.08	-180.65
		POEN-9-101.5	-0.18	-185.45
		POEN-55-182.1	-1.86	-194.25
		POEN-72-169.3	-7.02	-221.19
		POEN-34-118.1	-3.50	-241.47
		POEN-80-67.1	-0.08	-274.61
		POEN-14-124.2	-6.60	-277.43
		POEN-16-124.5	-8.70	-289.43
		POEN-19-24.4	-2.02	-295.17
		POEN-26-114.2	-2.35	-309.31
		POEN-94-30.1	-0.05	-309.41
		POEN-85-186.4	-1.02	-321.65
		POEN-65-192.1	-2.57	-353.34
		POEN-10-51.2	-9.75	-369.38
		POEN-59-179.1	-1.39	-377.73
		POEN-3-51.5	-8.54	-423.98
		POEN-32-45.2	-0.81	-430.21
		POEN-50-71.5	-0.13	-475.53
		POEN-93-61.5	-0.26	-481.41
		POEN-21-120.4	-3.62	-541.25
		POEN-15-92.3	-1.23	-606.44
		POEN-74-66.5	-3.56	-645.50
		POEN-2-51.4	-8.25	-653.22
		POEN-99-102.1	-1.93	-665.34
		POEN-28-192.5	-0.71	-677.12
		POEN-7-49.3	-9.50	-697.80
		POEN-5-49.5	-6.36	-787.89
		POEN-4-113.3	-8.04	-1089.65
Q3	Above-average NDM and above-average yield	POEN-71-196.2	2.81	700.00
		POEN-133-172.3	3.65	686.77
		POEN-107-104.2	3.02	681.42
		POEN-123-57.1	2.05	648.03
		POEN-104-143.4	2.35	593.72
		POEN-131-43.2	1.00	585.57
		POEN-136-115.5	1.72	567.76
		POEN-109-110.3	0.55	554.41
		POEN-150-199.1	2.47	507.76
		POEN-148-196.3	2.18	495.89
		POEN-79-183.3	1.00	488.86

POEN-145-115.1	2.06	487.95
POEN-86-29.1	1.42	474.16
POEN-37-175.3	3.28	465.21
POEN-121-10.3	0.55	450.17
POEN-142-104.1	3.44	442.17
POEN-126-188.1	1.72	436.65
POEN-89-143.1	0.97	422.02
POEN-84-43.3	1.97	414.21
POEN-125-57.2	1.63	405.53
POEN-17-108.3	1.08	356.17
POEN-111-195.4	2.44	331.04
POEN-24-3.5	0.50	319.25
POEN-122-178.4	3.82	308.77
POEN-56-14.5	2.18	274.17
POEN-39-62.3	1.08	253.56
POEN-118-119.3	0.97	253.38
POEN-43-189.1	0.58	244.52
POEN-22-103.1	2.23	240.18
POEN-40-77.1	3.11	233.14
POEN-45-66.1	0.38	226.40
POEN-141-135.3	0.08	179.70
POEN-116-178.2	2.44	168.51
POEN-115-43.4	1.00	167.28
POEN-120-43.5	0.71	166.35
POEN-83-56.1	0.34	160.19
POEN-103-194.1	1.01	155.59
POEN-112-57.3	3.31	152.51
POEN-54-125.5	0.38	147.07
POEN-98-188.5	0.66	140.37
POEN-91-176.3	1.93	137.37
POEN-53-162.1	0.59	133.35
POEN-51-32.1	1.60	132.38
POEN-102-43.1	1.22	119.53
POEN-108-178.1	0.97	116.67
POEN-92-200.1	1.72	113.69
POEN-144-195.3	1.64	111.87
POEN-128-130.5	0.87	108.07
POEN-82-176.4	1.42	105.27
POEN-134-188.3	0.59	90.43
POEN-42-52.2	4.45	81.96
POEN-66-94.1	1.34	75.81
POEN-101-149.1	0.17	69.96
POEN-52-170.1	0.17	68.21
POEN-138-57.4	1.81	65.15
POEN-119-166.4	0.08	49.72

		POEN-87-186.3	0.50	43.65
		POEN-78-197.5	2.02	41.93
		POEN-110-71.1	2.06	41.27
		POEN-117-129.5	1.93	27.83
		POEN-67-165.5	2.06	21.11
Q4	Above-average NDM and below-average yield	POEN-130-103.2	0.79	-8.00
		POEN-1-3.1	1.34	-19.25
		POEN-139-153.1	0.87	-22.15
		POEN-70-52.4	2.39	-56.98
		POEN-146-178.5	3.02	-72.86
		POEN-76-110.4	0.80	-78.81
		POEN-90-30.3	0.34	-80.53
		POEN-147-197.1	1.84	-81.02
		POEN-38-14.3	0.24	-89.06
		POEN-73-189.3	0.24	-99.06
		POEN-33-113.2	2.35	-108.28
		POEN-41-189.2	0.03	-116.20
		POEN-96-141.2	1.00	-117.51
		POEN-149-195.1	0.80	-154.81
		POEN-132-195.5	0.38	-162.58
		POEN-36-14.4	1.01	-174.25
		POEN-62-29.3	1.97	-193.76
		POEN-30-35.4	2.44	-207.78
		POEN-129-35.5	2.18	-222.85
		POEN-97-158.2	1.13	-225.25
		POEN-135-188.2	2.89	-237.59
		POEN-69-52.1	5.41	-272.98
		POEN-11-78.1	0.97	-287.62
		POEN-127-56.5	3.65	-295.32
		POEN-124-103.3	2.06	-305.39
		POEN-29-191.5	0.16	-334.68
		POEN-31-29.5	1.81	-334.92
		POEN-88-52.5	1.51	-355.23
		POEN-140-179.2	1.84	-384.26
		POEN-105-196.1	1.30	-399.25
		POEN-44-191.4	0.38	-411.12
		POEN-137-29.2	1.43	-450.93
		POEN-106-30.5	1.13	-483.05
		POEN-143-30.2	0.35	-505.01

Table S2. Distribution of inbred lines according to BLUPs for number of days to maturity (NDM) and grain yield (GY) across four performance quadrants (Q1–Q4) for Population E3. Quadrants represent different agronomic profiles relative to the population mean: Q1 (below–average NDM and above–average yield), Q2 (below–average NDM and below–average yield), Q3 (above–average NDM and above–average yield), and Q4 (above–average NDM and below–average yield).

Quadrant	Agronomic class	Inbred line	BLUP NDM	BLUP yield
Q1	Below-average NDM and above-average yield	BR245BR278-5-42.1	-0.76	557.68
		BR245BR278-55-3.2	-2.41	295.51
		BR245BR278-34-68.5	-4.97	294.62
		BR245BR278-45-3.1	-3.64	262.57
		BR245BR278-48-68.3	-1.60	257.00
		BR245BR278-49-75.4	-4.67	227.94
		BR245BR278-47-38.5	-3.85	211.53
		BR245BR278-2-71.3	-4.84	152.49
		BR245BR278-7-69.3	-4.15	147.49
		BR245BR278-69-41.3	-1.79	143.87
		BR245BR278-53-4.3	-0.98	136.90
		BR245BR278-9-10.3	-6.83	136.05
		BR245BR278-10-42.3	-2.20	130.72
		BR245BR278-59-53.3	-0.57	129.41
		BR245BR278-30-38.3	-3.65	100.22
		BR245BR278-26-23.2	-2.00	77.73
		BR245BR278-4-11.5	-9.22	77.60
		BR245BR278-13-20.4	-5.59	77.41
		BR245BR278-51-34.5	-0.14	77.26
		BR245BR278-20-6.5	-5.80	73.96
		BR245BR278-18-71.1	-2.63	60.67
		BR245BR278-40-9.1	-1.17	51.99
		BR245BR278-29-11.1	-6.53	22.36
		BR245BR278-17-3.4	-5.87	15.84
		BR245BR278-68-5.4	-1.26	7.93
		BR245BR278-41-16.1	-3.87	2.74
Q2	Below-average NDM and below-average yield	BR245BR278-35-57.2	-0.14	-23.28
		BR245BR278-43-21.2	-0.11	-27.38
		BR245BR278-46-41.2	-3.44	-34.05
		BR245BR278-24-3.3	-5.67	-45.41
		BR245BR278-3-69.1	-5.18	-51.23
		BR245BR278-11-23.1	-0.16	-53.93
		BR245BR278-63-41.4	-2.17	-97.72
		BR245BR278-25-29.2	-3.32	-102.79
		BR245BR278-79-68.1	-0.44	-113.89
		BR245BR278-44-16.2	-2.71	-131.97
		BR245BR278-74-3.5	-2.00	-174.92
		BR245BR278-28-1.2	-1.58	-205.86
		BR245BR278-15-71.2	-1.60	-207.21

		BR245BR278-39-5.2	-1.39	-208.12
		BR245BR278-8-24.2	-1.79	-233.56
		BR245BR278-32-47.1	-1.39	-294.47
		BR245BR278-6-11.2	-9.30	-474.50
Q3	Above-average NDM and above-average yield	BR245BR278-37-38.4	2.74	814.48
		BR245BR278-33-68.4	2.57	247.65
		BR245BR278-21-21.3	1.34	245.60
		BR245BR278-70-17.3	4.22	202.89
		BR245BR278-65-41.5	0.48	185.24
		BR245BR278-19-23.4	0.27	148.61
		BR245BR278-62-12.1	5.42	135.60
		BR245BR278-27-17.1	1.34	120.26
		BR245BR278-16-34.3	1.75	114.26
		BR245BR278-60-55.3	5.53	110.87
		BR245BR278-73-52.3	0.67	89.97
		BR245BR278-22-21.4	0.68	87.77
		BR245BR278-61-14.5	0.87	71.96
		BR245BR278-57-74.5	9.33	48.59
		BR245BR278-12-15.2	6.77	44.35
		BR245BR278-52-6.2	1.09	25.15
		BR245BR278-78-52.2	7.48	24.33
		BR245BR278-66-74.1	1.75	23.79
Q4	Above-average NDM and below-average yield	BR245BR278-54-12.4	3.56	-17.33
		BR245BR278-31-17.4	2.65	-64.08
		BR245BR278-72-74.2	1.49	-76.13
		BR245BR278-42-6.1	0.47	-115.58
		BR245BR278-38-38.2	3.57	-121.12
		BR245BR278-77-74.4	6.28	-124.90
		BR245BR278-1-57.1	6.23	-151.66
		BR245BR278-67-57.4	5.04	-162.25
		BR245BR278-23-59.3	2.52	-164.92
		BR245BR278-14-23.5	1.54	-175.10
		BR245BR278-36-1.1	2.93	-183.58
		BR245BR278-76-35.5	4.92	-222.91
		BR245BR278-75-52.4	5.22	-233.62
		BR245BR278-58-10.1	8.63	-263.20
		BR245BR278-50-12.3	4.84	-277.13
		BR245BR278-64-34.1	0.88	-288.74
		BR245BR278-56-57.3	3.60	-309.09
		BR245BR278-71-35.1	5.01	-488.37
		BR245BR278-80-55.5	9.74	-550.85

Table S3. Coefficients of determination (R^2) and results of the type III Wald test for fixed effects in the mixed models fitted for NDF, NRD, and NDM in two soybean populations, considering two environmental covariates: accumulated precipitation and mean temperature. Jaboticabal, SP.

Population	Trait	Environmental covariate	Conditional R^2	Marginal R^2	β coefficient	SE	95% CI	χ^2	*p-Value*	n
E2	NDF	Accumulated precipitation	0.91	0.56	0.420	0.015	0.259 to 0.318	838.47	< 0.001***	718
		Mean temperature	0.87	0.30	-2.289	0.138	-2.37 to -1.828	260.26	< 0.001***	
	NRD	Accumulated precipitation	0.81	0.13	0.052	0.008	0.057 to 0.088	65.74	< 0.001***	717
		Mean temperature	0.79	0.22	-3.502	0.303	-4.329 to -3.142	142.92	< 0.001***	
	NDM	Accumulated precipitation	0.94	0.18	0.101	0.009	0.083 to 0.118	126.58	< 0.001***	717
		Mean temperature	0.93	0.32	-5.565	0.334	-6.22 to -4.91	277.33	< 0.001***	
E3	NDF	Accumulated precipitation	0.97	0.65	0.169	0.015	0.139 to 0.2	120.75	< 0.001***	439
		Mean temperature	0.96	0.68	-2.587	0.219	-3.016 to -2.158	139.57	< 0.001***	
	NRD	Accumulated precipitation	0.89	0.11	-0.068	0.02	-0.107 to -0.029	11.44	< 0.001***	439
		Mean temperature	0.91	0.21	4.867	0.67	3.554 to 6.18	52.77	< 0.001***	
	NDM	Accumulated precipitation	0.96	0.35	-0.004	0.014	-0.031 to 0.023	0.08	0.783 ns	439
		Mean temperature	0.96	0.35	-0.203	0.581	-1.341 to 0.935	0.12	0.726 ns	

χ^2: Chi-square; p-value: significance of fixed effects in each experiment; NDF: number days to flowering; NRD: number of reproductive days; NDM: number of days to maturity; SE: standard error of the estimated beta coefficient; 95% CI: 95% confidence interval for the estimated beta coefficient; n: represents the number of observations used to fit each covariate model after removing missing values. Conditional and marginal R^2 values were estimated using the performance package. β coefficients were extracted from the fixed-effect estimates of the mixed models. p-values refer to type III Wald tests for the environmental covariates. Significance codes: *** *p*-Value ≤ 0.001; ** *p*-Value ≤ 0.01; * *p*-Value ≤ 0.05; *p*-Value ≤ 0.1; ns: not significant (*p*-Value > 0.1).

Table S4. Decomposition of the coefficient of determination into fixed and random effects in mixed models fitted for phenological traits in two soybean populations. Jaboticabal, SP.

Population	Trait	Environmental covariate	Model R^2	Fixed R^2	Random R^2
E2	NDF	Accumulated precipitation	0.918	0.320	0.597
		Mean temperature	0.867	0.275	0.592
	NRD	Accumulated precipitation	0.798	0.147	0.651
		Mean temperature	0.775	0.239	0.535
	NDM	Accumulated precipitation	0.930	0.183	0.746
		Mean temperature	0.921	0.340	0.581
E3	NDF	Accumulated precipitation	0.965	0.672	0.293
		Mean temperature	0.957	0.704	0.254
	NRD	Accumulated precipitation	0.876	0.105	0.771
		Mean temperature	0.885	0.088	0.797
	NDM	Accumulated precipitation	0.950	0.380	0.571
		Mean temperature	0.950	0.384	0.566

Model R^2, Fixed R^2 and Random R^2 were obtained using the rsq package. NDF: number of days to flowering; NRD: number of reproductive days; NDM: number of days to maturity.

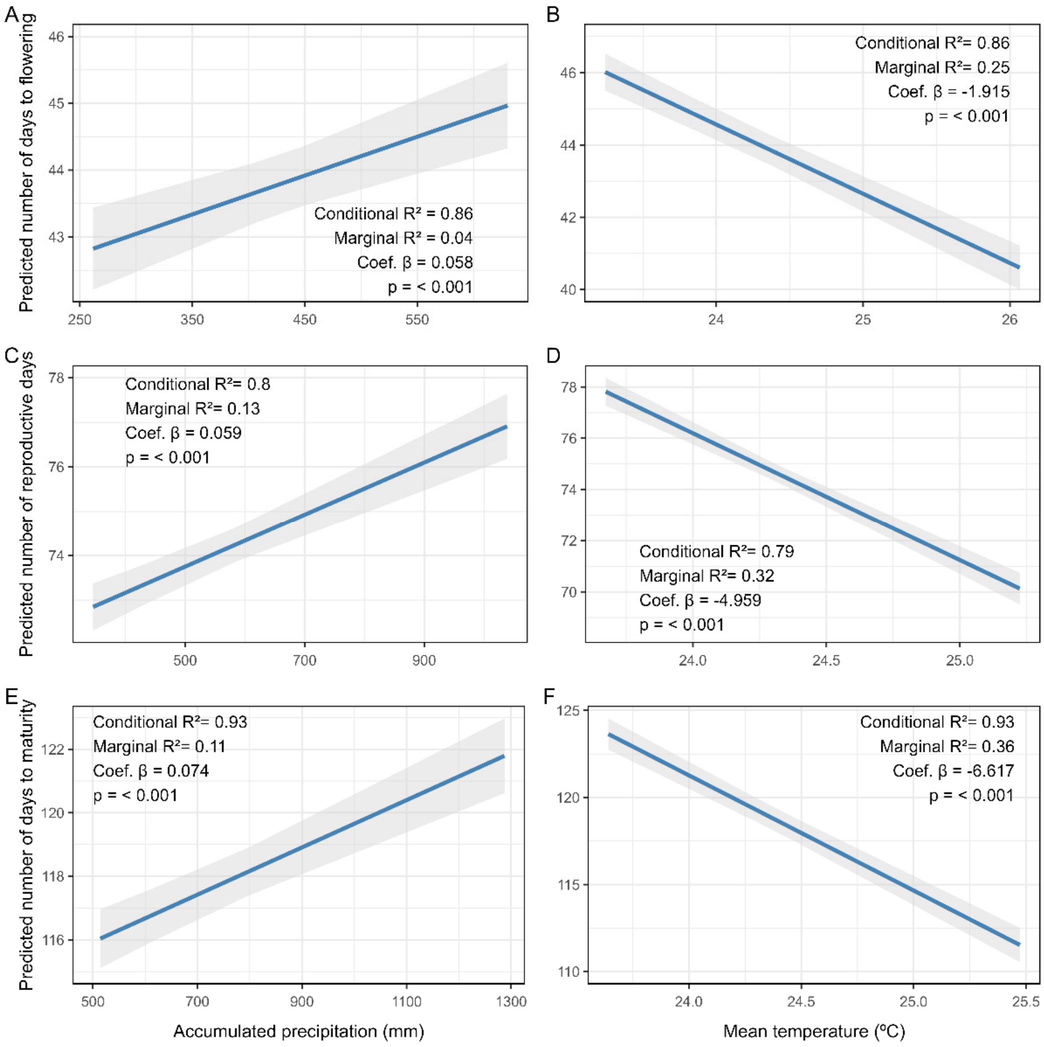

Figure S2. Marginal predictions of NDF, NRD, and NDM obtained from mixed models adjusted with accumulated precipitation and mean temperature calculated using fixed post-sowing environmental windows as fixed covariates for Population E2 : (A) NDF as a function of accumulated precipitation; (B) NDF as a function of mean temperature; (C) NRD as a function of accumulated precipitation; (D) NRD as a function of mean temperature; (E) NDM as a function of accumulated precipitation; and (F) NDM as a function of mean temperature. Blue lines represent the model-adjusted predictions, and the shaded areas represent the corresponding 95% confidence intervals. The fitted relationships represent statistical associations and should not be interpreted as evidence of causal effects. Jaboticabal, SP.

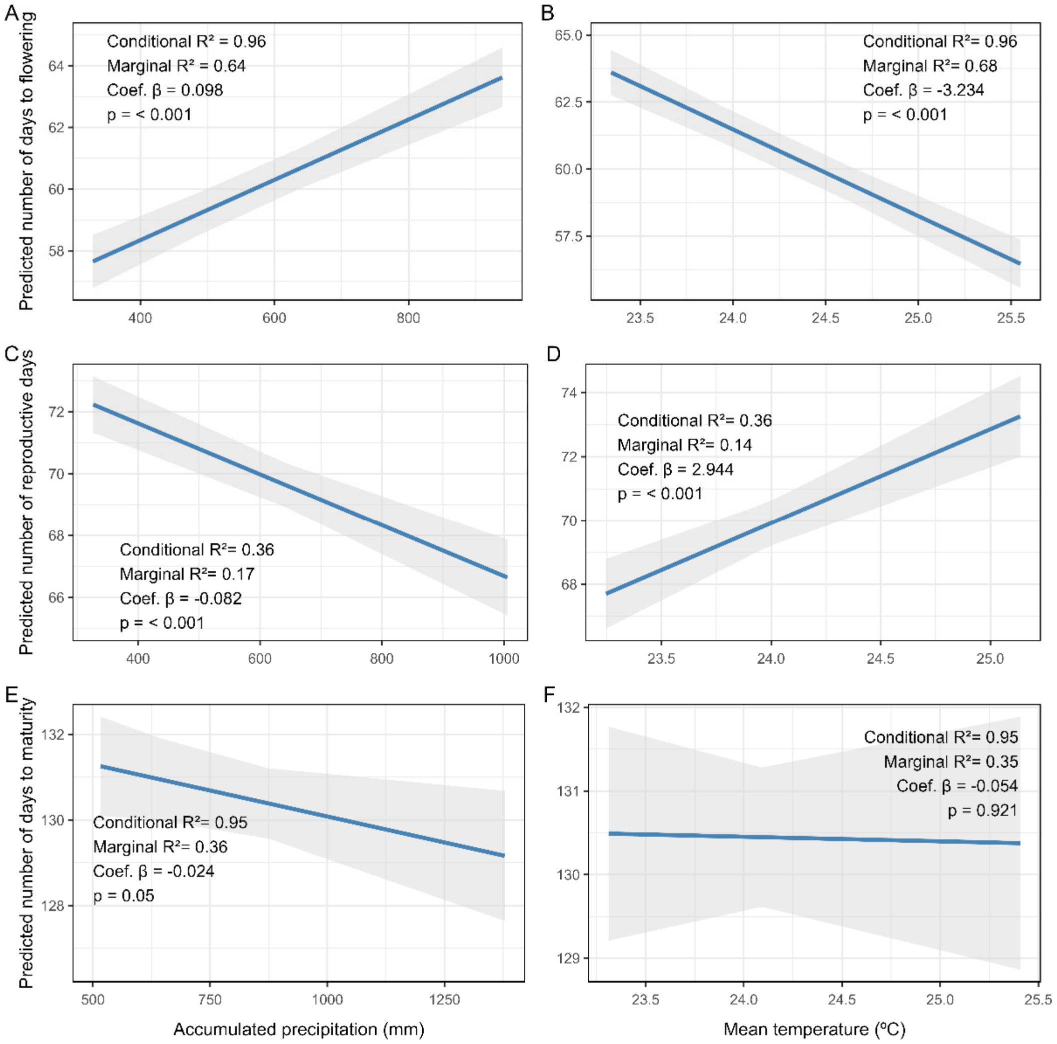

Figure S3. Marginal predictions of NDF, NRD, and NDM obtained from mixed models adjusted with accumulated precipitation and mean temperature calculated using fixed post-sowing environmental windows as fixed covariates for Population E3: (A) NDF as a function of accumulated precipitation; (B) NDF as a function of mean temperature; (C) NRD as a function of accumulated precipitation; (D) NRD as a function of mean temperature; (E) NDM as a function of accumulated precipitation; and (F) NDM as a function of mean temperature. Blue lines represent the model-adjusted predictions, and the shaded areas represent the corresponding 95% confidence intervals. The fitted relationships represent statistical associations and should not be interpreted as evidence of causal effects. Jaboticabal, SP.

Table S5. Coefficients of determination (R^2) and results of the type III Wald test for fixed effects in mixed models fitted for NDF, NRD, and NDM in two soybean populations using accumulated precipitation and mean temperature calculated from fixed post-sowing environmental windows as covariates. Jaboticabal, SP.

Population	Trait	Environmental covariate	Conditional R^2	Marginal R^2	β coefficient	SE	95% CI	χ^2	*p-Value*	n
E2	NDF	Accumulated precipitation	0.86	0.04	0.058	0.012	0.034 to 0.082	22.83	< 0.001***	718
		Mean temperature	0.86	0.25	-1.915	0.134	-2.177 to -1.653	205.69	< 0.001***	
	NRD	Accumulated precipitation	0.8	0.13	0.059	0.007	0.045 to 0.073	66.17	< 0.001***	717
		Mean temperature	0.79	0.32	-4.959	0.307	-5.56 to -4.358	261.45	< 0.001***	
	NDM	Accumulated precipitation	0.93	0.11	0.074	0.009	0.056 to 0.093	61.88	< 0.001***	717
		Mean temperature	0.93	0.36	-6.617	0.362	-7.327 to -5.907	333.78	< 0.001***	
E3	NDF	Accumulated precipitation	0.96	0.64	0.098	0.011	0.077 to 0.119	85.23	< 0.001***	439
		Mean temperature	0.96	0.68	-3.234	0.273	-3.769 to -2.699	140.29	< 0.001***	
	NRD	Accumulated precipitation	0.36	0.17	-0.082	0.012	-0.106 to -0.059	46.63	< 0.001***	439
		Mean temperature	0.36	0.14	2.944	0.503	1.958 to 3.93	34.25	< 0.001***	
	NDM	Accumulated precipitation	0.95	0.36	-0.024	0.012	-0.049 to 0	3.84	0.050 ns	439
		Mean temperature	0.95	0.35	-0.054	0.545	-1.123 to 1.014	0.01	0.921 ns	

χ^2: Chi-square; p-value: significance of fixed effects in each experiment; NDF: number days to flowering; NRD: number of reproductive days; NDM: number of days to maturity; SE: standard error of the estimated beta coefficient; 95% CI: 95% confidence interval for the estimated beta coefficient; n: represents the number of observations used to fit each covariate model after removing missing values. Conditional and marginal R^2 values were estimated using the performance package. β coefficients were extracted from the fixed-effect estimates of the mixed models. p-values refer to type III Wald tests for the environmental covariates. Significance codes: *** *p*-Value ≤ 0.001; ** *p*-Value ≤ 0.01; * *p*-Value ≤ 0.05; *p*-Value ≤ 0.1; ns: not significant (*p*-Value > 0.1).
